# Obinutuzumab‐induced serum sickness following salvage therapy for chronic lymphocytic leukemia

**DOI:** 10.1002/ccr3.969

**Published:** 2017-04-24

**Authors:** Julie Saba, Aaron C. Logan

**Affiliations:** ^1^ Division of Malignant Hematology and Blood and Marrow TransplantationUniversity of CaliforniaSan FranciscoCalifornia

**Keywords:** Chronic lymphocytic leukemia, monoclonal antibody, obinutuzumab, serum sickness

## Abstract

The incidence of serum sickness following treatment of CLL with obinutuzumab has not been fully characterized, but is likely rare. Consideration should be given to this diagnosis in appropriate circumstances so that effective corticosteroid therapy can be initiated to alleviate inflammatory symptoms and organ dysfunction in a timely manner.

## Introduction

Obinutuzumab is a humanized monoclonal antibody that recognizes the CD20 antigen present on all mature B cells. In 2013, it was approved by the Food and Drug Administration (FDA) for treatment of chronic lymphocytic leukemia (CLL) and was the first drug to receive the FDA's new breakthrough therapy designation. Although a number of adverse effects associated with its usage have been reported, there are no documented cases of serum sickness occurring in response to the use of obinutuzumab. Herein, we report a patient who developed serum sickness on two separate occasions after receiving the drug for treatment of Stage IV CLL. As obinutuzumab usage increases, it is likely that additional cases of serum sickness will be observed, and clinicians should be aware of this potential complication.

## Case Details

A 55‐year‐old female with stage IV CLL, who 10 weeks prior to presentation had begun a course of obinutuzumab, presented to the emergency room with acute pleuritic chest and back pain. Her history included CLL diagnosis 2 years prior to presentation, treatment with a combination of rituximab and lenalidomide on a clinical trial, which was discontinued after 1 year due to inadequate response demonstrated by persistent replacement of 90% of the bone marrow with CLL. The patient's CLL cells exhibited trisomy 11 on conventional cytogenetics, which was corroborated by amplification of the 11q23 locus as assessed by fluorescent in situ hybridization analysis, an abnormality which had been present since diagnosis. At the time of presentation, the patient's white blood cell count revealed mild leukopenia at 2.6 x 10^9^/L (3.4–10 x 10^9^/L normal), and she demonstrated no significant lymphadenopathy or organomegaly. The primary indication for treatment was the presence of persistent thrombocytopenia.

Thereafter she initiated treatment with single‐agent obinutuzumab. Prior to this presentation, obinutuzumab had been administered on four occasions and was well tolerated except for mild type IV hypersensitivity reactions (mild, self‐resolving urticarial rashes). Three days prior to presentation, the patient received a fifth dose of obinutuzumab and developed a temperature of 38C during the infusion. On the day of presentation, the patient reported the sudden onset of severe, constant, diffuse pain in the chest and upper back. Physical examination was unremarkable, with normal vital signs and no evidence of rash, arthritis, angioedema, or organomegaly. EKG, urinalysis, serum chemistry panel, lactate dehydrogenase, and serum uric acid level were normal. Troponin I was 0.03 (<0.05 normal). Due to unrelenting pain of unclear etiology, additional studies were performed in the emergency room including a chest CT with contrast that revealed small bilateral pleural effusions, no evidence of pulmonary embolism, and no other abnormalities. A thoracic spine MRI was unremarkable.

Over the subsequent 3 days the chest pain resolved, but the patient developed severe bilateral hip and left knee pain, generalized weakness and malaise, and bilateral paresthesias in her fingers and toes. Weakness progressed until she was unable to walk, stand, or move her right leg. Laboratory test results demonstrated inflammation with CRP 304 mg/L (<6.3 normal), ESR 89 mm/h (0–15 mm/h normal), C3 99 mg/dL (71–159 mg/dL normal), C4 21.2 mg/dL (13–30 mg/dL normal). Uric acid was normal at 4.5 mg/dL (2.9–6.6 mg/dL normal) and there was no biochemical evidence of tumor lysis syndrome. Of note, the patient had no prior history of arthritis, articular effusions, or autoimmunity. A presumptive diagnosis of serum sickness was made, and the patient started prednisone dosed at 1 mg/kg daily. Within 24 h her symptoms began rapidly to resolve, and she was discharged home on a gradual steroid taper.

Based on peripheral blood count monitoring, the patient's CLL remained stable for almost 4 months, after which her platelet and neutrophil count began to nadir, requiring intervention. The patient requested that obinutuzumab be retried due to her motivation to avoid treatment with cytotoxic therapy, and because oral small molecular inhibitors such as ibrutinib and idelalisib had not yet received FDA approval. The sixth obinutuzumab dose was split into a 100 mg test dose and 900 mg dose administered on two consecutive days, in combination with 1500 mg intravenous methylprednisolone daily for 3 days following a CLL treatment protocol combining high‐dose steroids with rituximab.^1^ The patient tolerated the infusions without incident; however, 5 days after the first infusion day, she returned to the hospital with severe (10 out of 10) chest and upper back pain, accompanied by bilateral paresthesias of fingers and toes. Her physical examination was unremarkable. She received hydromorphone, diphenhydramine, acetaminophen, and additional methylprednisolone and her pain resolved. Her C4 was below normal at 7.1 mg/dL, C3 85 mg/dL, ESR 26 mm/h, high sensitivity CRP 49.5 (normal is <3). There again was no biochemical evidence of tumor lysis syndrome. The patient was discharged from the clinic on 1.5 mg/kg prednisone daily, and all symptoms resolved within 4 days. Ten days later, the patient was asymptomatic and her C4 had normalized at 25.3 mg/dL. Steroids were weaned over 4 weeks without recurrent symptoms.

## Discussion

Serum sickness was not reported to be an adverse event in the clinical studies leading to the approval of obinutuzumab.^2^, ^3^, ^4^, ^5^, ^6^ It is thus likely to be a rare complication of treatment, as is also the case for rituximab, with a recent survey of the literature finding only 33 reported cases of rituximab‐induced serum sickness.^7^ Our patient experienced recurrent serum sickness upon reexposure to obinutuzumab, thus satisfying Koch's postulates regarding causality. This case demonstrates very well that serum sickness may present with variable symptoms, and laboratory findings such as complement depletion, may not be sensitive. Inflammatory markers were differentially elevated during the two episodes our patient experienced, with the first being accompanied by normal C3 and C4 levels, with markedly elevated ESR and CRP, and the second being associated with partial complement consumption and less markedly elevated inflammatory markers. High‐dose corticosteroid may have attenuated the rise in ESR and CRP, but failed to prevent recurrent serum sickness upon obinutuzumab reexposure. Continued corticosteroid therapy was effective at achieving relatively rapid resolution of symptoms, as is typically the case.

Our patient's experience is similar to that of patients developing serum sickness in response to rituximab therapy for autoimmune and neoplastic diseases.^7^ Serum sickness response to therapeutic molecules may be problematic for continuation of therapy, but is uniformly manageable with corticosteroid therapy at an initial dose equivalent to at least 1 mg/kg of recipient body weight daily, followed by taper over several weeks. Insufficient taper duration may be accompanied by symptomatic recurrence.

## Conclusions

We present a patient who exhibited serum sickness on two occasions with repeat exposure to obinutuzumab to treat refractory CLL. This adverse event was successfully treated with corticosteroids, as is typical for serum sickness. As obinutuzumab usage increases, it is likely that additional cases of serum sickness will be observed, thus clinicians should be aware of this potential complication so that the clinical syndrome can be treated promptly.

## Authorship

JS and ACL: wrote the manuscript.

## Conflict of Interest

The authors have no conflicts of interest to declare.
